# The association of low body mass index with neonatal morbidities in preterm infants

**DOI:** 10.1038/s41598-021-98338-5

**Published:** 2021-09-22

**Authors:** Byoung Kook Lee, Jun Hyeok Lee, Jeongmin Shin, Young Hwa Jung, Chang Won Choi

**Affiliations:** 1 Department of Pediatrics, Chungnam National University Sejong Hospital, Sejong, Republic of Korea; 2 Department of Biostatistics, Yonsei University Wonju College of Medicine, Wonju, Republic of Korea; 3 Department of Pediatrics, Seoul National University Bundang Hospital, 82 Gumi-ro, 173 Beon-gil, Bundang-gu, Seongnam-si, Gyeonggi-do 13620 Republic of Korea; 4 Department of Pediatrics, Seoul National University College of Medicine, Seoul, Republic of Korea

**Keywords:** Diseases, Health care, Medical research, Risk factors

## Abstract

Little is known about the association between body proportionality at birth and neonatal outcomes in preterm infants. Body mass index (BMI) is one of the weigh-for-length ratios that represent body proportionality. The objective of this study was to examine whether BMI at birth affects neonatal outcomes in preterm infants. We assessed 3115 preterm (< 30 weeks), very low birth weight (< 1500 g) infants born between January 2013 and December 2016 and registered in the Korean Neonatal Network database. Using gender-specific BMI for gestational age curves, z-scores of BMI at birth were calculated. Low-, normal-, and high-BMI were defined as BMI z-scores of less than − 1, from − 1 to 1, and greater than 1, respectively. Neonatal morbidities and mortality in low- and high-BMI groups were compared to those in normal-BMI group. The low-BMI group had an increased risk of bronchopulmonary dysplasia, bronchopulmonary dysplasia or death, and necrotizing enterocolitis after adjusting for baseline characteristics and the birth weight z-score. High-BMI group had comparable neonatal outcomes to those of normal-BMI group. Low BMI at birth was associated with an increased risk of bronchopulmonary dysplasia and necrotizing enterocolitis, whereas High BMI at birth was not associated with adverse neonatal outcomes.

## Introduction

Body mass index (BMI) is used as a parameter of adiposity in children and adults^1^. In adults, BMI has J-shaped associations with overall mortality and most cause-specific mortalities, with the lowest mortality at 25 kg/m^2^^2^. High BMI in early infancy is strongly associated with early childhood obesity^3^, and high childhood BMI is associated with type 2 diabetes, hypertension, and coronary heart disease in adulthood^4^. Simple anthropometric measurements, such as birth weight, length, and head circumference of newborn infants, provide information on body size, but not on body proportionality. The weight-to-length ratio can provide information on body proportionality in one parameter^5^. Gender-specific BMI for gestational age (GA) curves with Lamda-Mu-Sigma (LMS) values and percentiles were reported by Olsen et al. in 2015 based on data from 254,454 singleton preterm and term infants born in the United States who survived to discharge^6^. Olsen et al. considered BMI the best weight-to-length ratio, as it was most highly correlated with weight and uncorrelated with height both in boys (GA ≥ 24 weeks) and girls (GA ≥ 25 weeks)^6^.

Small for gestational age (SGA) poses additional vulnerability to prematurity and further adversely impacts neonatal outcomes in preterm infants^7,8^. However, it remains unclear how disproportionality between birth weight and length affects neonatal outcomes in preterm infants. BMI is one of the weight-for-length ratios that represent body proportionality^6^. However, little is known about the association between BMI at birth and neonatal outcomes in preterm infants. In this study, we aimed to examine the impact of BMI at birth on neonatal morbidities and mortality during the admission to neonatal intensive care unit (NICU) using a nationwide cohort of very low birth weight (VLBW) infants.

## Methods

**Figure 1:**
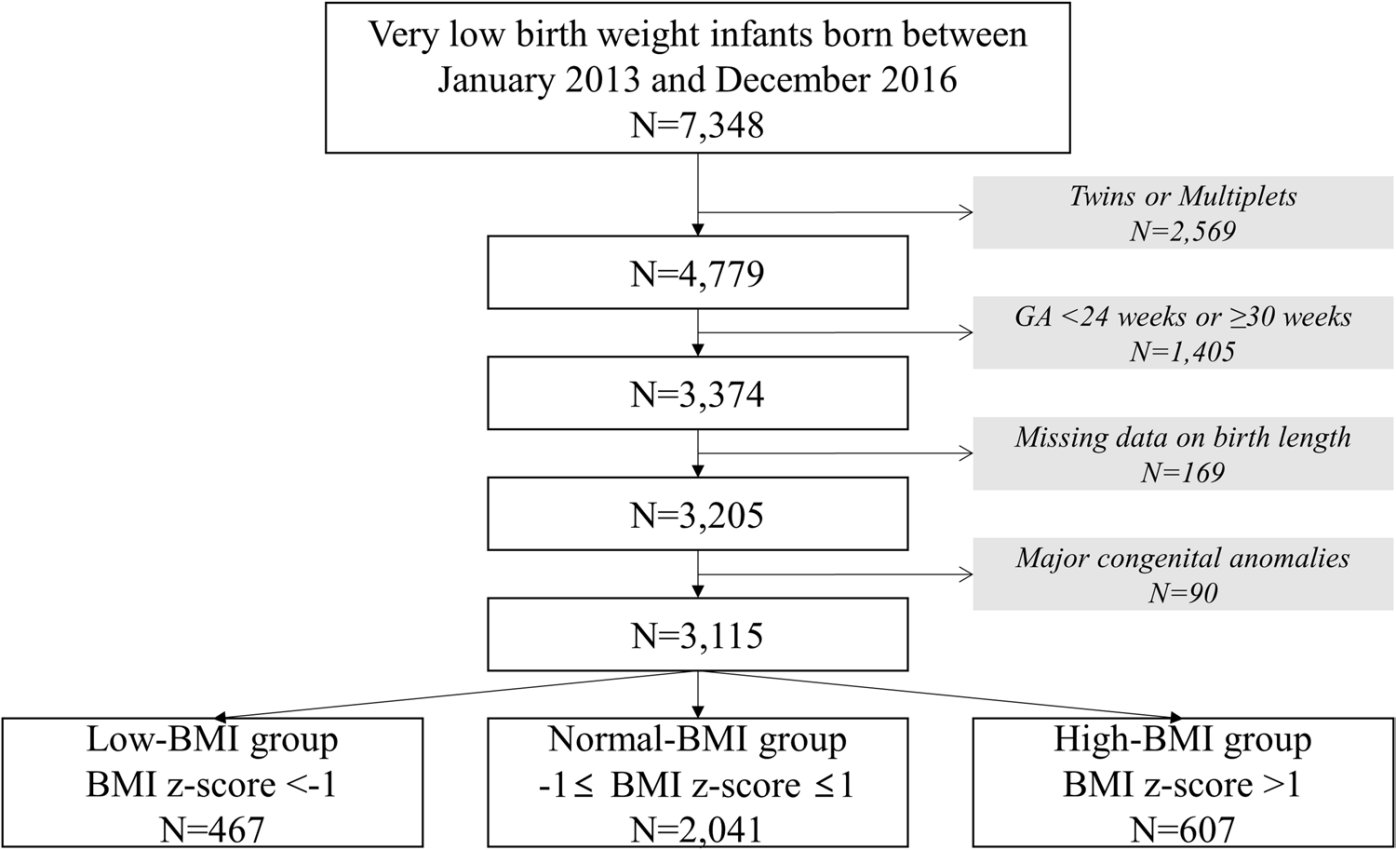
Flow diagram of the study population. *GA* gestational age; *BMI* body mass index.

We used a prospective cohort of VLBW infants of the Korean Neonatal Network (KNN), a nationwide VLBW infant registry in which 70 NICUs across the South Korea participate^9^. For this study, 7348 VLBW (< 1500 g) infants born between January 2013 and December 2016 who were enrolled. Of these 7348 VLBW infants, 2569 infants who were twins or high-order multiplets, 1405 infants born < 24 weeks GA or ≥ 30 weeks GA, 169 infants whose data on length at birth were missing, and 90 infants with major congenital anomalies were excluded. Finally, 3115 infants were analysed. Based on BMI z-score, these 3115 VLBW infants were divided into a low-BMI group (z-score < -1, n = 467), normal-BMI group (-1 ≤ z-score ≤ 1, n = 2041), and high-BMI group (z-score > 1, n = 607) as shown in Fig. 1. BMI was calculated as (weight in gram/[length in centimetre]^2^) × 10. BMI z-score at birth was calculated using the LMS values of the gender-specific BMI for GA curves by Olsen et al.^6^. Z-scores in this study were calculated by GA in completed weeks, as LMS data of Olsen et al.’s BMI for GA curves were based on GA in completed weeks, not in weeks^+days^.

The KNN registry collects maternal, delivery, and neonatal information of VLBW infants admitted to the NICU^9^. Baseline characteristics include maternal age, gestational diabetes mellitus (GDM), preeclampsia, histologic chorioamnionitis, antenatal corticosteroids, mode of delivery, gender, GA, birth weight, length, head circumference at birth of infants. Neonatal morbidities include respiratory distress syndrome (RDS), high-grade (≥ grade III) intraventricular haemorrhage (IVH), necrotizing enterocolitis (NEC), retinopathy of prematurity (ROP) requiring LASER or vebacizumab treatment, periventricular leukomalacia (PVL), bronchopulmonary dysplasia (BPD), and mortality during the admission to NICU. Regarding antenatal corticosteroid administration, only cases in which antenatal corticosteroid course was completed within 7 days before delivery were included. RDS was defined as respiratory insufficiency that manifested at or shortly after birth, accompanied by typical radiologic findings compatible with RDS, and required surfactant replacement therapy. BPD was defined as the need for supplementary oxygen at 36 weeks postmenstrual age (PMA). Severe BPD was defined as the need for ≥ 30% oxygen and/or positive pressure at 36 weeks PMA^10^. NEC was defined according to the modified Bell’s criteria, and only stages ≥ II were included^11^. PVL was defined as the detection of the presence of a periventricular cyst upon cranial ultrasound or brain magnetic resonance imaging. Mortality was defined as the death which occurred during the initial admission to NICU.

Categorical variables were presented as numbers and percentages, and continuous variables were presented as the mean ± SD. Categorical variables were analysed using the Chi-square test or Fisher’s exact test. Continuous variables were analysed using an independent two-sample t-test. Multivariate logistic regression analyses were performed with variables which were significant (*P*-value < 0.1) in the univariate analyses included in the logistic regression models to confirm the independent associations between BMI z-score at birth and neonatal morbidities and mortality. The results were expressed as odds ratios (ORs) and 95% confidence intervals (CIs). *P*-values of < 0.05 were considered statistically significant. The statistical analysis was performed by SAS software version 9.4 (SAS, Cary, USA).

### Ethics statements

This study was approved by the institutional review board of Chungnam National University Sejong hospital (CNUSH 2020–11-006) and the Korean Neonatal Network (2018–016). All methods were performed in accordance with the ethical standards of our institutional research committee and with the 1964 Helskinki declaration and its later amendments.

### Ethics committee/institutional review board

The Korean Neonatal Network registry was approved by the institutional review board and informed consent was obtained from parents upon enrolment at each participating hospital.

### Consent for publication

All authors agreed to publish this manuscript.

## Results

### Baseline characteristics

The distributions of birth weight, length, BMI, and BMI z-score at birth of the 3115 infants included in the study are presented in Supplementary Fig. 1A–D. While the birth weight had a slightly skewed distribution toward higher birth weights, the length and BMI at birth showed normal distributions, and their means ± SD were 1008 ± 254 g, 35.5 ± 3.4 cm and 7.9 ± 1.2, respectively. The distributions of BMI and BMI z-score across the GA are shown in Supplementary Fig. 1E, F. There were poor correlations between GA and BMI at birth and BMI z-score at birth. There were 1633 boys and 1482 girls.

The baseline characteristics of each BMI group are presented in Table 1. There were no significant differences in GA among the low-BMI group, normal-BMI group, and high-BMI group. When compared to infants in the normal-BMI group, those in the low-BMI group had lower birth weights (762 ± 229 g *vs.* 1028 ± 233 g, *P* < 0.001), and those in the high-BMI group had higher birth weights (1129 ± 211 g *vs.* 1028 ± 233 g, *P* < 0.001). The ratio of girls to boys did not differ significantly among the three BMI groups. Of the maternal and delivery characteristics, preeclampsia was more common in the low-BMI group and less common in the high-BMI group than in the normal-BMI group. Caesarean section delivery was more common in the low-BMI group than in the normal-BMI group. On the other hand, histologic chorioamnionitis was less common in the low-BMI group and more common in the high-BMI group than in the normal-BMI group. There were no significant differences in maternal age, rate of GDM, or coverage of antenatal corticosteroids among the three BMI groups.

**Table 1:** Baseline characteristics of each BMI group.

	Normal-BMI group n = 2041	Low-BMI group n = 467	*P*-value	High-BMI group n = 607	*P*-value
Maternal age (years)	32.8 ± 4.5	33.4 ± 4.5	0.16	32.9 ± 4.4	0.83
Gestational diabetes	179 (8.8)	33 (7.1)	0.27	56 (9.2)	0.75
Preeclampsia	274 (13.4)	222 (47.5)	< 0.001	42 (6.9)	< 0.001
Histologic chorioamnionitis	931 (47.3)	123 (30.1)	< 0.001	265 (52.6)	0.04
Antenatal steroids	1005 (60.1)	244 (64.0)	0.32	270 (57.2)	0.59
Caesarean section	1383 (67.8)	392 (83.9)	< 0.001	388 (63.9)	0.09
Male gender	1079 (52.9)	241 (51.6)	0.64	313 (51.6)	0.58
Gestational age (weeks)	27.0 ± 2.9	27.1 ± 2.8	0.52	26.9 ± 3.3	0.19
Birth weight (g)	1028 ± 233	762 ± 229	< 0.001	1129 ± 211	< 0.001
Birth weight z-score	0.22 ± 0.71	-1.07 ± 0.85	< 0.001	0.83 ± 0.65	< 0.001
Birth length (cm)	36.0 ± 3.1	34.1 ± 4.2	< 0.001	34.7 ± 3.0	< 0.001
Birth length z-score	0.28 ± 0.98	-0.46 ± 1.62	< 0.001	-0.22 ± 0.99	< 0.001
BMI at birth	7.81 ± 0.69	6.38 ± 0.69	< 0.001	9.37 ± 1.39	< 0.001

Data are expressed as n (%), mean ± SD.

### Associations between BMI at birth and neonatal morbidities and mortality

**Figure 2:**
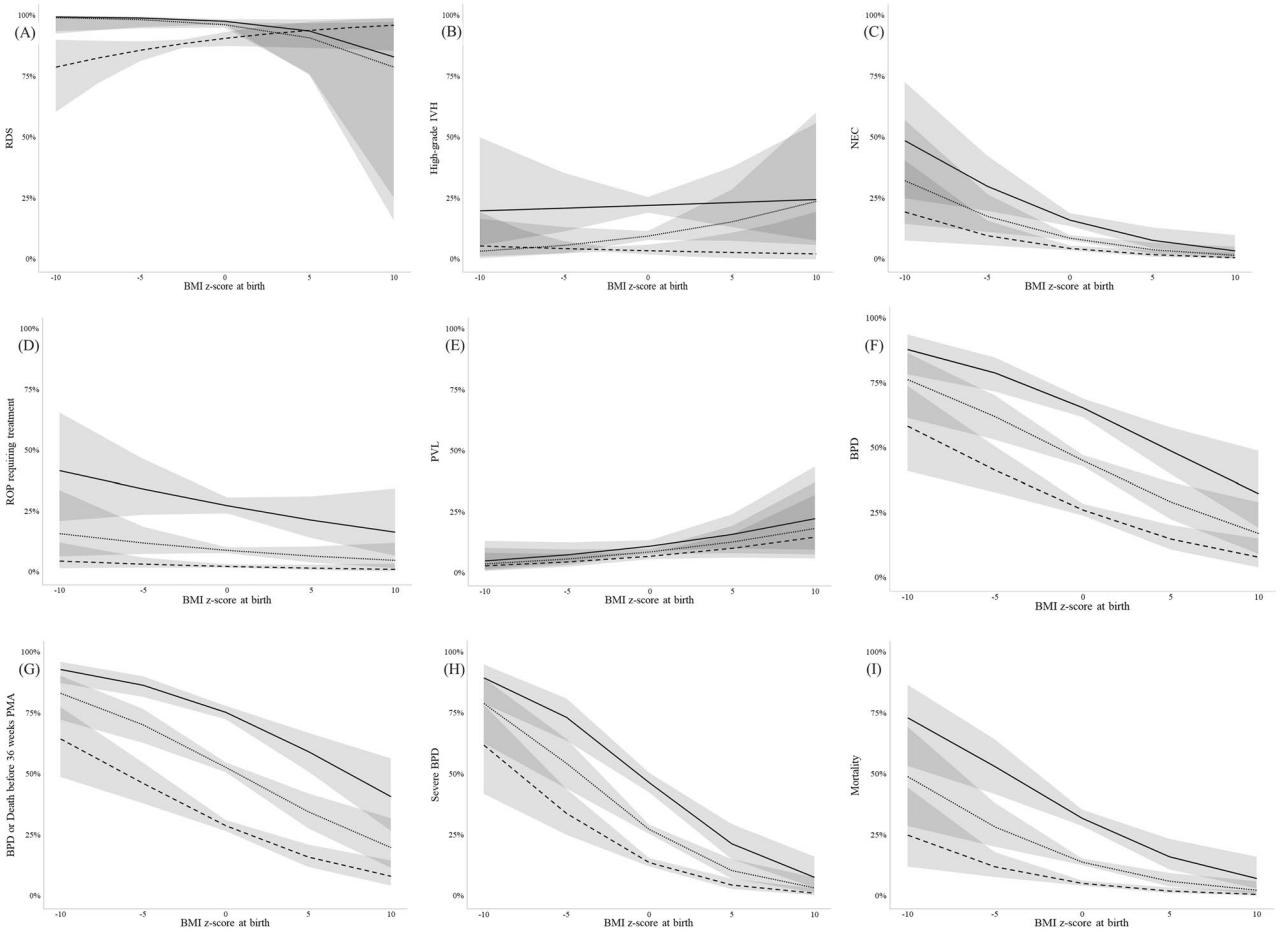
Predicted probability of neonatal morbidities and mortality according to BMI z-score at birth according to gestational age group. Regression lines and their 95% confidence intervals (gray shades) are shown for each gestational age group. Solid lines, 24–25 weeks gestational age group; dotted lines, 26–27 weeks gestational age group; dashed lines, 28–29 weeks gestational age group. *RDS* respiratory distress syndrome; *BMI* body mass index; *IVH* intraventricular hemorrhage; *NEC* necrotizing enterocolitis; *ROP* retinopathy of prematurity; *PVL* periventricular leukomalacia; *BPD* bronchopulmonary dysplasia; *PMA* postmenstrual age.

**Figure 3:**
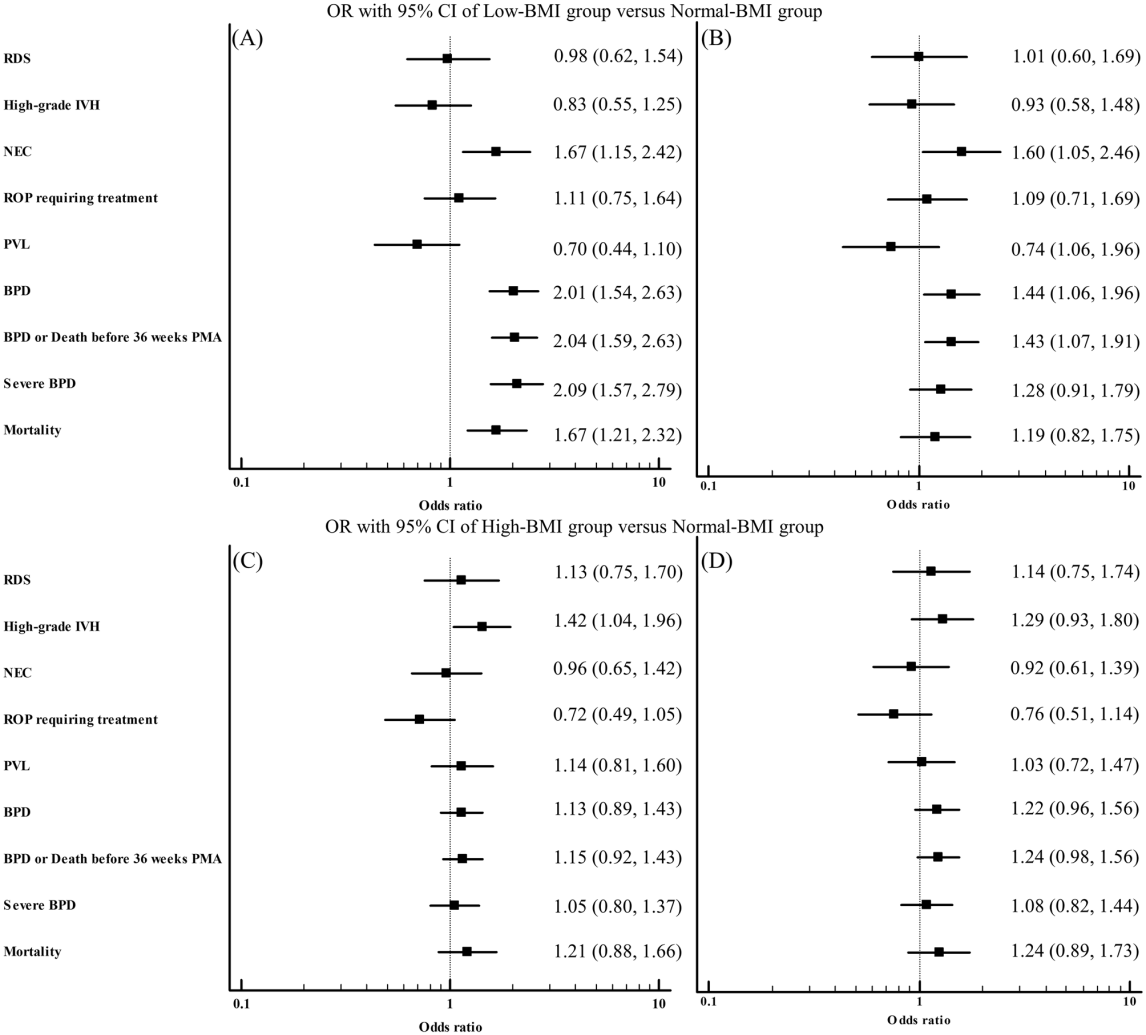
Adjusted odds ratios and their 95% confidence intervals for neonatal morbidities and mortality of (**A**,**B**) the low-BMI group and (**C**,**D**) the high-BMI group compared to the normal-BMI group. Adjustment was made for preeclampsia, histologic chorioamnionitis, caesarean section delivery, and gestational age (**A**,**C**). Further adjustment was made for birth weight z-score (**B**,**D**). *OR* odds ratio; *CI* confidence interval; *BMI* body mass index; *RDS* respiratory distress syndrome; *IVH* intraventricular hemorrhage; *NEC* necrotizing enterocolitis; *ROP* retinopathy of prematurity; *PVL* periventricular leukomalacia; *BPD* bronchopulmonary dysplasia; *PMA* postmenstrual age.

The rates of neonatal morbidities and mortality according to BMI z-score at birth are shown for each GA group in Fig. 2. When compared to the normal-BMI group, the low-BMI group had more NEC, more BPD, more BPD or death before 36 weeks PMA, more severe BPD, and higher mortality. The rates of RDS, high-grade IVH, ROP requiring treatment, and PVL did not differ significantly between the low-BMI group and the normal-BMI group (Table 2). After adjusting for baseline characteristics, including preeclampsia, histologic chorioamnionitis, caesarean section delivery, and GA, low BMI was significantly associated with increased risks of BPD, BPD or death before 36 weeks PMA, severe BPD, NEC, and higher mortality (Fig. 3A). After further adjustment for birth weight z-score, low BMI was significantly associated with increased risks of BPD, BPD or death before 36 weeks PMA, and NEC. (Fig. 3B).

**Table 2:** Comparisons of neonatal morbidities and mortality between low- and normal-BMI groups and between high- and normal-BMI groups.

	Normal-BMI group n = 2041	Low-BMI group n = 467	*P-*value	High-BMI group n = 607	*P-*value
RDS	1901 (93.1)	437 (93.9)	0.76	572 (94.2)	0.35
High-grade IVH	203 (9.9)	42 (9.0)	0.55	72 (11.9)	0.19
NEC (≥ stage II)	164 (8.1)	61 (13.2)	0.001	44 (7.3)	0.55
ROP requiring treatment	214 (10.5)	52 (11.1)	0.74	53 (8.7)	0.22
PVL	181 (9.1)	30 (6.6)	0.08	57 (9.7)	0.69
BPD	665 (37.0)	203 (52.3)	< 0.001	202 (37.9)	0.72
BPD or death before 36 weeks PMA	907 (44.4)	282 (60.4)	< 0.001	275 (45.3)	0.71
Severe BPD	394 (21.9)	143 (36.9)	< 0.001	116 (21.8)	0.95
Mortality	263 (12.9)	98 (21.0)	< 0.001	79 (13.0)	0.95

Data are expressed as n (%), mean ± SD.

There were no significant differences in the rates of neonatal morbidities or mortality between the high-BMI group and the normal-BMI group (Table 2). After adjusting for baseline characteristics, including preeclampsia, histologic chorioamnionitis, caesarean section delivery, and GA, high BMI was significantly associated with increased risk of high-grade IVH. Otherwise, there were no significant associations between high BMI and neonatal morbidities or mortality. (Fig. 3C). After further adjustment for birth weight z-score, high BMI was not significantly associated with any neonatal morbidities or mortality. (Fig. 3D).

## Discussion

Fetal growth restriction (FGR) leads to a variety of short-term and long-term adverse outcomes in newborn infants, especially in those born preterm^8^. Frequently FGR is used interchangeably with SGA in clinical practice, although a portion of infants with FGR would fall under the appropriate-for-gestational age (AGA) category^12^. The concept of body proportionality in the newborn period and early infancy for assessing the risk of childhood obesity has been gaining attention. Roy et al. suggested that BMI in early infancy is a better predictor of childhood obesity than weight for length, which is the current standard for assessing body proportionality in the first 2 years of life recommended by the American Academy of Pediatrics^3,13^. However, little is known about the association between BMI at birth and neonatal outcomes in preterm infants. In this study, low BMI at birth was associated with increased risks of BPD and NEC, regardless of birth weight for GA. The rates of RDS, high-grade IVH, PVL, ROP requiring treatment, and mortality were not associated with low BMI after adjustment for covariates, including birth weight z-score. In order to assess the association of BMI with neonatal outcomes independently from the impact of birth weight for GA, we further included birth weight z-score in the multivariate logistic regression models. Despite good correlation between BMI z-score and birth weight z-score, no significant multicollinearity was found between them throughout the analyses.

The association of low BMI at birth with increased risks of BPD and NEC is our key finding. Regarding BPD, low BMI at birth was significantly associated with BPD and BPD or death before 36 weeks PMA. BPD is frequently complicated by pulmonary hypertension, poor somatic growth, and adverse neurological outcomes^14^. Preterm SGA infants are 45% more likely to develop BPD or die from respiratory complications after birth than preterm AGA infants^15^. They also need more prolonged mechanical ventilation than preterm AGA infants^16^. To the best of our knowledge, no study has evaluated the association between body proportionality at birth and BPD. In the present study, low BMI at birth remained significantly associated with BPD and BPD or death before 36 weeks PMA after further adjustment for birth weight z-score. These result suggests that BPD is associated with body disproportionality at birth regardless of whether the preterm infant is SGA or AGA.

Low BMI may represent reduced fat mass or reduced fat-free mass, or both. It has been known that large deposition of fat tissue occurs during the third trimester of gestation^17^. In a recent study of body composition in newborn infants using air displacement plethysmography, both fat mass and fat-free mass gradually increased as GA increased, from 34 to 42 weeks^18^. However, the rate of increase was higher in fat mass than in fat-free mass during the same period. In the same study, wasted newborn infants with low BMI (< 3rd percentile) had a lower body fat percentage (4.0% *vs.* 10.4%) and lower fat mass/fat-free mass ratio (4.3% *vs.* 11.8%) than non-wasted infants with normal BMI^18^. These findings suggest that low BMI in newborns represents a greater reduction in body fat rather than fat-free mass. Because most fat is located in subcutaneous tissue and is not intra-abdominal in newborn infants^19^, infants with low BMI are likely to have a lean body shape. However, these data were obtained from term or late-preterm infants, and thus may not be applicable to the interpretation of our results. Our study population comprised preterm infants born before 30 weeks GA, when the deposition of fat tissue is not in earnest yet. Therefore, low BMI in our study may suggest low fat-free mass rather than low fat mass.

Another finding of our study was the association between low BMI at birth and an increased risk of NEC. Similar to BPD, NEC is more likely to develop in preterm infants with FGR^20^. Splanchnic blood flow can be reduced in FGR due to redistribution of cardiac output to the vital organs. Reduced splanchnic blood flow and ensuing gut hypoxia/ischemia may contribute to the development of NEC^21^. However, little is known about the association between body disproportionality and NEC. Because NEC also continued to be significantly associated with low BMI at birth after adjustment for birth weight z-score, it seems that body disproportionality exposes preterm infant to an increased risk of developing NEC independently from the risk from FGR as in BPD.

In a population-based cohort study of adults, high BMI above 25 kg/m^2^ was associated with an increased mortality^2^. In a systematic review by Park et al., there was evidence of associations between high BMI in childhood and type 2 diabetes, hypertension, and coronary heart disease^4^. However, most of these associations were not independent from adult BMI. In the present study, high BMI at birth was not significantly associated with any neonatal morbidities or mortality. This result suggests that high BMI in preterm infant does not indicate an unhealthy condition unlike adults and older children.

We used a prospective cohort of VLBW infants of the KNN registry for this study. Because the KNN registry enrols VLBW infants regardless of GA, the KNN VLBW infant cohort had a biased high number of SGA or low-BMI preterm infants born at relatively later GAs. To minimize this bias in the KNN cohort, we only included VLBW infants born earlier than 30 weeks GA in this study. As shown in Supplementary Fig. 1F, BMI z-scores have an even distribution from 24 to 30 weeks GA.

In this study, we defined a low or high BMI as a BMI z-score at birth of < -1 or > 1. The appropriate cut-off value for low or high BMI at birth is not known yet. When more stringent criteria for low or high BMI at birth (BMI z-score at birth of < -2 or > 2) was employed, the size difference between low or high BMI group and normal BMI group was too big to be compared without statistical bias due to unequal variance of imbalanced data^22^.

The ethnic homogeneity of our cohort may be considered a strength of the present study, as ethnic differences may affect BMI at birth. However, there were some limitations to our study. Firstly, our cohort data do not include information on parental anthropometric measurements or maternal nutritional status, which might have affected the BMI of the offspring. Secondly, information on potential postnatal modulators of BPD, such as respiratory management strategies and nutritional status, was not available in our cohort data, and thus, adjustment for these factors was not made. Thirdly, we calculated BMI z-scores using the BMI curves for preterm infants by Olsen et al.^6^, which were based on the United States population. Although no information was provided on the ethnic composition of their study population, there will be clear ethnic difference between theirs and out study population. Calculating BMI z-score with BMI curves based on different ethnic population is another weak point of our study.

In conclusion, we found that low BMI at birth was associated with increased risks of BPD and NEC independently from the effect of SGA in preterm infants. To our knowledge, this is the first study to investigate the association between BMI at birth and neonatal outcomes in preterm infants. Body disproportionality seems to expose preterm infants to additional risk for adverse neonatal outcomes. Future study will be needed to reveal an abnormal body composition responsible for increased risks of BPD and NEC and underlying mechanisms leading to these morbidities.

## Supplementary Information

Supplementary Information 1.

## Data Availability

This work was supported by the Research Program funded by the Korea Centers for Disease Control and Prevention. There are ethical restrictions on sharing a deidentified data set unless permitted by the CDC of Korea. Data availability was subjected to the Act on Bioethics and Safety [Law No. 1518, article 18 (Provision of Personal Information)]. Contact for sharing the data or access the data can be possible only through the data committee of Korean neonatal network (http://knn.or.kr) and after permitted by the CDC of Korea. Detail contact information was as follows: data access committee; Yun Sil Chang (yschang@skky.edu), ethics committee; So-Young Kim (sykimped@catholic.ac.kr).

## Abbreviations

BMI: Body mass index
SGA: Small for gestational age
GA: Gestational age
BPD: Bronchopulmonary dysplasia
PMA: Postmenstrual age
aOR: Adjusted odds ratio
NEC: Necrotizing enterocolitis
FGR: Fetal growth restriction
NICU: Neonatal intensive care unit
VLBW: Very low birth weight
KNN: Korean Neonatal Network
GDM: Gestational diabetes mellitus
RDS: Respiratory distress syndrome
IVH: Intraventricular hemorrhage
ROP: Retinopathy of prematurity
PVL: Periventricular leukomalacia
PMA: Postmenstrual age
SD: Standard deviation
SGA: Small for gestational age
AGA: Appropriate for gestational age
