# Supplementary figures and images for: The association of low body mass index with neonatal morbidities in preterm infants
Source: Sci Rep. 2021 Sep 22;11:18841. doi: 10.1038/s41598-021-98338-5 (PMC8458459; doi:10.1038/s41598-021-98338-5)

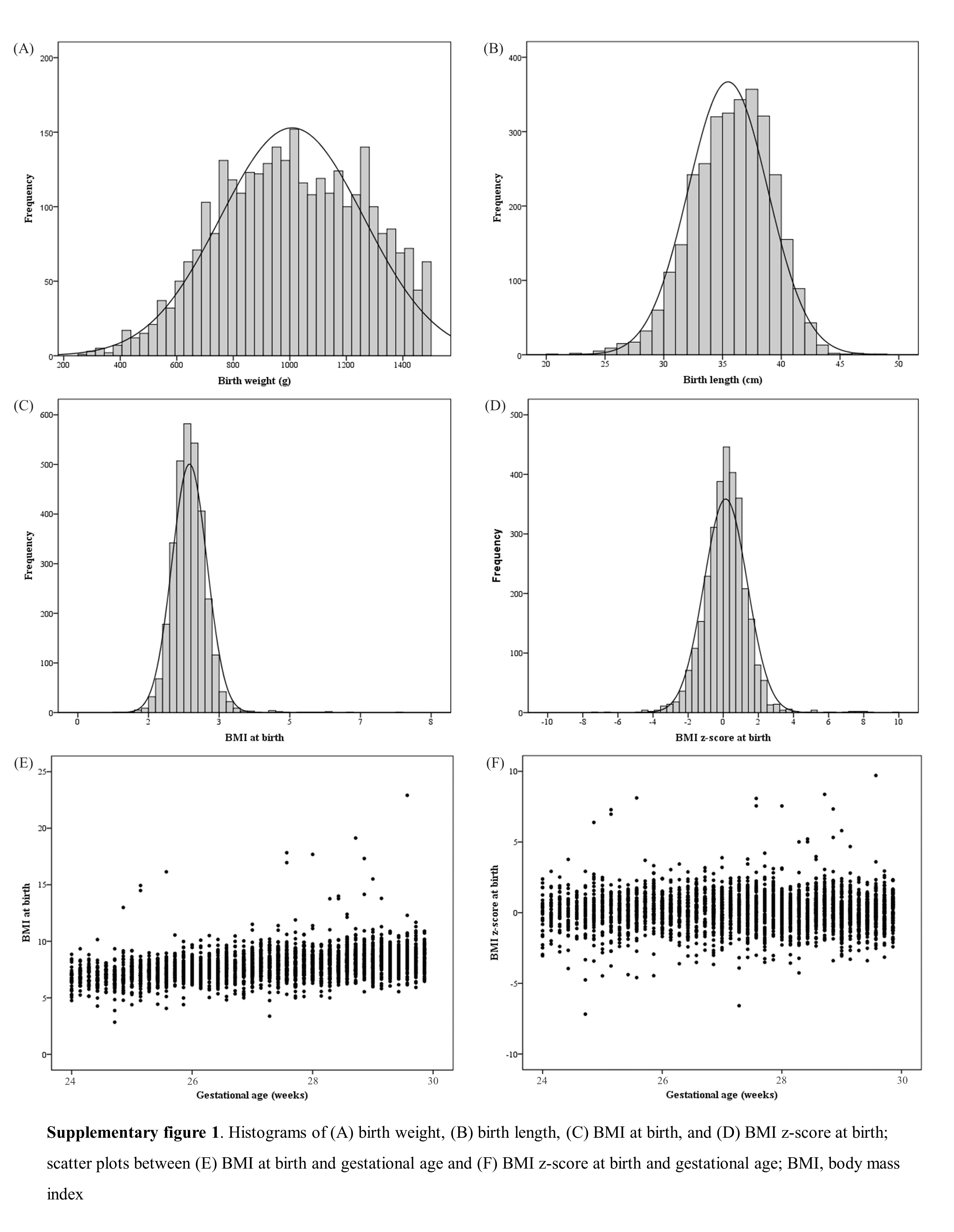

Supplement: Supplementary file 1 — Supplementary Information 1. [file 41598_2021_98338_MOESM1_ESM.tif]
